# Viability of Total Ammoniacal Nitrogen Recovery Using a Polymeric Thin-Film Composite Forward Osmosis Membrane: Determination of Ammonia Permeability Coefficient

**DOI:** 10.3390/polym16131834

**Published:** 2024-06-27

**Authors:** Shirin Shahgodari, Joan Llorens, Jordi Labanda

**Affiliations:** Department of Chemical Engineering and Analytical Chemistry, University of Barcelona, Martí i Franquès 1, 08028 Barcelona, Spain; shirin.shahgodari@ub.edu (S.S.); jllorensl@ub.edu (J.L.)

**Keywords:** membrane technology, forward osmosis, nutrient recovery, modeling, ammonia permeability coefficient

## Abstract

Total ammoniacal nitrogen (TAN) occurs in various wastewaters and its recovery is vital for environmental reasons. Forward osmosis (FO), an energy-efficient technology, extracts water from a feed solution (FS) and into a draw solution (DS). Asymmetric FO membranes consist of an active layer and a support layer, leading to internal concentration polarization (ICP). In this study, we assessed TAN recovery using a polymeric thin-film composite FO membrane by determining the permeability coefficients of NH_4_^+^ and NH_3_. Calculations employed the solution–diffusion model, Nernst–Planck equation, and film theory, applying the acid–base equilibrium for bulk concentration corrections. Initially, model parameters were estimated using sodium salt solutions as the DS and deionized water as the FS. The NH_4_^+^ permeability coefficient was 0.45 µm/s for NH_4_Cl and 0.013 µm/s for (NH_4_)_2_SO_4_ at pH < 7. Meanwhile, the NH_3_ permeability coefficient was 6.18 µm/s at pH > 9 for both ammonium salts. Polymeric FO membranes can simultaneously recover ammonia and water, achieving 15% and 35% recovery at pH 11.5, respectively.

## 1. Introduction

Wastewaters are complex fluids made up of organic and inorganic compounds, including valuable molecules like ammonium salts [1]. For simplicity, total ammoniacal nitrogen (TAN) is defined as the sum of both the ammonium ion and ammonia. The ammonia/ammonium ratio is determined by the acid–base equilibrium that has a pKa value of 9.24. TAN is found in various types of wastewaters, including domestic, municipal, industrial, and livestock streams [2]. Treatment methods often focus on TAN removal using chemical processes like chlorination and electrochemical oxidation [3,4], biological processes like ammonium enzymatic conversion, and bioelectrochemical systems [5,6]. Physical processes like ammonia stripping, adsorption, ion exchange, and reverse osmosis are also used for TAN recovery [7,8].

Forward osmosis (FO) and gas permeation membrane contactors, which are promising membrane technologies, offer energy-efficient ways to recover TAN. Their low energy consumption makes them attractive options for sustainable wastewater treatment. In both processes, the transfer of the molecules through the membrane wall is controlled by the chemical concentration gradient between the two phases or solutions in contact with the membrane [9]. Gas permeation membrane contactors use both hydrophobic and hydrophilic membranes, although most membranes are hydrophobic in nature in order to remove gaseous molecules such as NH_3_ and CO_2_ [10]. These membranes prevent the permeation of water molecules and allow the diffusion of ammonia into a trapping acidic solution, where the ammonia is converted into an ammonium salt fertilizer [11]. Several studies have evaluated the recovery of ammonia from supernatants generated by anaerobic digestion [11], livestock manure [12] and biowaste fermentation broths [13], reaching high recovery values (>80%). 

FO is mainly used to concentrate the feed solution, enabling water molecules to permeate through the membrane due to the osmotic pressure difference between the feed solution (FS) and the draw solution (DS). FO membranes have a unique structure consisting of an active layer responsible for the transport of selected substances and a porous support layer that provides a stable structure. FO membranes can operate in the FO mode, where the active layer is in contact with the FS, and in the PRO mode, where the active layer is in contact with the DS [14].

The water flux across the FO membrane is influenced by both the type and concentration of the draw solute, as well as the internal concentration polarization (ICP) occurring within the support layer of the membrane [15]. An effective draw solute must primarily be capable of generating a high osmotic pressure while minimizing the reverse solute flux [16]. The ICP is due to the movement of water and solute molecules through the membrane support. When the active layer is in contact with the FS, the water flux dilutes the solute concentration in the support (dilutive ICP), while the convective flux pushes the solute molecules in the FS into the support (concentrative ICP) when the active layer is in contact with the DS [15]. Mathematically, the ICP can be evaluated by the structural parameter, which depends on the physical features of the membrane support and the diffusivity coefficient of the solute [17]. In addition to the ICP, external concentration polarization (ECP) can play an important role in water permeation, as the ECP can reduce the effective driving force, thereby decreasing the water flux [18]. 

In addition, FO membranes allow the permeation of TAN (ammonium ion and ammonia), which can be present in the FS. Therefore, they can concentrate the FS, leading to the recovery of water and TAN in the DS. In an acidic environment (pH < 7), the ammonium ion is the predominant specie of TAN that can interact with the negatively charged membrane surface and move across the membrane [19,20]. However, its permeability is low and the recovery value in the DS is also low. Therefore, most research studies have focused on the rejection of the ammonium ion in the FS. For instance, human urine is a wastewater with a high concentration of TAN. It was reported to show an ammonium ion rejection of 50–60% at pH 6 that goes up to 90% at pH 3 [21,22]. However, ammonium ion rejection can be improved by modifying other factors. Almoalimi et al. (2022) found that the permeation of the ammonium ion can be reduced by minimizing the cation exchange with the draw solutes [23]. In addition, ammonium ion rejection can be improved through surface modification, thereby reducing the negative charge on the membrane. Bao et al. (2019) found an increase in the rejection from 40% of the pristine membrane to 86% of the grafted membrane [19]. 

The ratio of ammonia/ammonium ion content increases as the solution pH increases through the addition of chemicals, enhancing the permeation of TAN. Ammonia is a neutral molecule with a similar size to that of the water molecule and can diffuse easily across FO membranes. Therefore, ammonia can be recovered at a pH above 9 [24]. However, only a few studies have analyzed the use of FO membranes for simultaneous ammonia and water recovery in a process with low energy consumption. Ammonia recovery was studied in hydrolyzed urine that has a high ammonia content, showing a recovery value of around 50% [25]. Ray et al. (2024) achieved a recovery rate of 86% through strategic pH manipulation by keeping the DS pH < 6.5 and the FS pH > 11 [26]. 

The aim of this study was to contribute to the use of FO in the ammonia recovery process by determining the permeability coefficient of the ammonium ion and ammonia for a commercial FO membrane. To the best of our knowledge, these permeability coefficients have not been determined in any study to date. The performance of the FO membrane was evaluated with the solution–diffusion model in combination with the Nernst–Planck equation for flux inside the ICP and with the film theory quantifying the ECP. The model was completed by considering the acid–base equilibrium of the ammonium ion to correct the bulk concentrations. First, the permeation of different draw solutions formed of sodium salts was studied to determine the water permeability coefficient, the structural parameters of the membrane support layer (to evaluate the ICP), and the mass transfer coefficients of the ECP. After that, the ammonium ion permeability coefficient was estimated by fitting the experimental data at pH values lower than 7. As the pH increases with the addition of NaOH, the ammonia content increases and the solution becomes a mixture of the ammonium ion, ammonia, NaCl, and NaOH. Therefore, for a rigorous determination of the ammonia permeability coefficient, the contribution of all the solutes in the permeation process was considered.

## 2. Theory

### 2.1. Water Flux

The water flux, $J_{w}$, is defined as the product of the water permeability coefficient, $L_{p}$, and the driving force, which is calculated by the difference in the global osmotic pressure at the membrane wall in the DS, $\pi_{w,D}$, and FS, $\pi_{w,F}$:

(1) $$J_{w}=L_{p}\;\left(\pi_{w,D}-\pi_{w,F}\right)$$

It is assumed that the reflection coefficient is close to unity for all solutes. The global osmotic pressure is calculated by the sum of the osmotic pressure of all the species present in the solution:

(2) $$\pi_{w,Y}=\sum_{s=1}^{N_{Y}}\pi_{w,Y,s}=\sum_{s=1}^{N_{Y}}\emptyset_{w,Y,s}\;C_{w,Y,s}\;R_{m}\;T$$

where the subindex “*Y*” can be substituted by “*D*” or “*F*” to indicate the global osmotic pressure, solute concentration, and the number of species in the DS or in the FS, respectively. Equation (2) assumes the non-ideality of solutions and the osmotic pressure of each species can be calculated by the osmotic coefficient at the membrane wall, $\emptyset_{w,Y,s}$, the concentration at the membrane wall, $C_{w,Y,s}$, the ideal gas constant, and temperature. 

### 2.2. Reverse Solute Flux

**Figure 1:**
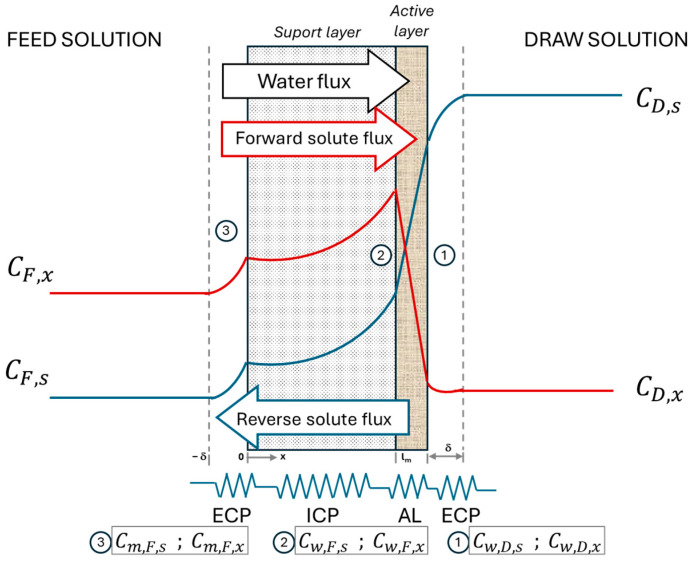
Schematic representation of the ICP and ECP in the PRO mode of operation. Number 1 refers to ECP at the DS, number 2 to ICP and number 3 to ECP at the FS.

The reverse solute flux, $J_{s}$, refers to the movement of a solute present in the DS to the FS and it is the opposite of the water flux (Figure 1). The reverse solute flux is defined as the product of the solute permeability coefficient, $L_{s}$, and the difference in solute concentration in the DS and FS:

(3) $$J_{s}=L_{s}\;\left(C_{w,D,s}-C_{w,F,s}\right)$$

The two solute concentrations at the membrane wall must be determined by a differential mole balance in the ECP layer and in the ICP in the support layer inside the membrane. These equations depend on the orientation of the membrane active layer, although only the equations for the active layer in contact with the DS were considered in this work. 

In the dilute ECP at the DS side, the evolution of the reverse solute flux inside the ECP layer can be expressed, following the Nernst–Planck equation, as:

(4) $$J_{s}=-J_{w}\;C_{D,s}\left(x\right)+\frac{d\left(D_{D,s}\;C_{D,s}\left(x\right)\right)}{dx}$$

where $D_{D,s}$ is the solute diffusion coefficient in the DS, which can be assumed to be a constant value, and $C_{D,s}\left(x\right)$ is the concentration polarization layer. The integration of Equation (4) can be achieved using the following initial and boundary conditions:

$$C_{D,s}\left(0\right)=C_{w,D,s};\;C_{D,s}\left(\delta \right)=C_{D,s}$$

where *δ* is the boundary layer thickness, leading to a simple relationship for the reverse solute concentration in the corresponding solution:

(5) $$C_{w,D,s}=C_{D,s}\;exp\left(-\frac{J_{w}}{k_{D}}\right)+\frac{J_{s}}{J_{w}}\;\left[exp\left(-\frac{J_{w}}{k_{D}}\right)-1\right]$$

where $k_{D}$ is the solute mass transfer coefficient at the DS side and is defined as $k_{D}=D_{D,s}/\delta$. 

Similar equations can be deduced for the dilutive ECP at the FS site:

(6) $$J_{s}=-J_{w}\;C_{F,s}\left(x\right)+\frac{d\left(D_{F,s}\;C_{F,s}\left(x\right)\right)}{dx}$$

where $D_{F,s}$ is the solute diffusion coefficient in the FS, which can also be assumed to be a constant value. Through the integration of Equation (6), using the initial condition ($C_{F,s}\left(0\right)=C_{m,F,s}$) and the boundary condition ($C_{F,s}\left(-\delta \right)=C_{F,s}$), the solute concentration at the FS side can be calculated as follows:

(7) $$C_{m,F,s}=C_{F,s}\;exp\left(\frac{J_{w}}{k_{F}}\right)+\frac{J_{s}}{J_{w}}\;\left[exp\left(\frac{J_{w}}{k_{F}}\right)-1\right]$$

where $k_{F}$ is the solute mass transfer coefficient at the DS side and is defined as $k_{F}=D_{F,s}/\delta$.

Inside the membrane support layer, the reverse solute flux can be expressed as:

(8) $$J_{s}=-J_{w}\;C_{F,s}\left(x\right)+\frac{d\left(D_{F,p,s}\;C_{F,s}\left(x\right)\right)}{dx}$$

where $D_{F,p,s}$ is the solute diffusion coefficient in the porous layer for the FS, which can be related to the bulk solute diffusion coefficient by considering the support porosity and tortuosity as $D_{F,p,s}=\varepsilon \;D_{F,s}/\tau$. Integrating Equation (8) across the support layer thickness, $l_{m}$, with the initial ($C_{F,s}\left(0\right)=C_{m,D,s}$) and boundary conditions ($C_{F,s}\left(l_{m}\right)=C_{w,F,s}$), the solute concentration at the membrane wall can be determined as:

(9) $$C_{w,F,s}=C_{F,s}\;exp\left(\;J_{w}\;\left(\frac{1}{k_{F}}+\frac{S}{D_{s}}\right)\right)+\frac{J_{s}}{J_{w}}\;\left[exp\left(J_{w}\;\left(\frac{1}{k_{F}}+\frac{S}{D_{s}}\right)\right)-1\right]$$

where *S* is the structural parameter of the support layer and is defined as $S=l_{m}\tau /\varepsilon$. Equations (5) and (9) relate the solute concentration at the membrane wall and the bulk solute concentration in the corresponding solution through the solute and water fluxes. The correlation of the bulk solute concentration of the two solutions with both fluxes can be expressed by the mole balance equations, which calculate the number of moles in the solution from the initial number of moles as follows:

(10) $$C_{F,s}\;\left(V_{F,o}-J_{w}\;A_{M}\;t\right)=C_{F,s,o}\;V_{F,o}+J_{s}\;A_{M}\;t$$

(11) $$C_{D,s}\;\left(V_{D,o}+J_{w}\;A_{M}\;t\right)=C_{D,s,o}\;V_{D,o}-J_{s}\;A_{M}\;t$$

where the subindex “*o*” identifies the initial values at time zero, $V_{D}$ and $V_{F}$ are the volumes of the draw and feed chambers, respectively, and $A_{M}$ is the effective membrane area. Note that the number of moles in both solutions are time-dependent, like the volume and the solute concentrations. 

Assuming that the reverse solute flux can be calculated from Equation (3) at any time and by combining with Equations (5) and (9)–(11), an individual equation of the solute concentration in the bulk and at the membrane walls of both solutions can be calculated from the water and solute permeabilities, water flux, structural parameter, and mass transfer coefficients. At this point, the osmotic pressure at the membrane wall of each species and the water flux can be calculated from Equations (1) and (2).

### 2.3. Forward Solute Flux

The direct solute flux, $J_{x}$, refers to the movement of a solute present in the FS to the DS as follows (Figure 1):

(12) $$J_{x}=L_{x}\;\left(C_{w,F,x}-C_{w,D,x}\right)$$

As shown in Figure 1, the direct solute flux is in the same direction as the water flux and, thus, the ECP at the FS side is a concentrative process. The solute molecules are more retained than the water molecules and the solute concentration increases at the feed membrane layer. The concentrative ECP can be expressed as follows:

(13) $$J_{x}=J_{w}\;C_{F,x}\left(x\right)-\frac{d\left(D_{F,x}\;C_{F,x}\left(x\right)\right)}{dx}$$

By integrating Equation (13), using the initial condition ($C_{F,x}\left(0\right)=C_{m,F,x}$) and the boundary condition ($C_{F,x}\left(-\delta \right)=C_{F,x}$), and assuming a constant solute diffusion coefficient, the solute concentration at the FS side can be calculated as follows:

(14) $$C_{m,F,x}=C_{F,x}\;exp\left(\frac{J_{w}}{k_{F}}\right)+\frac{J_{x}}{J_{w}}\;\left[1-exp\left(\frac{J_{w}}{k_{F}}\right)\right]$$

In the case of the active layer in contact with the DS, the solute concentration is concentrated across the membrane support and the diffusive transport moves in the opposite direction to that of the convective movement. The concentrative concentration polarization can be expressed as:

(15) $$J_{x}=J_{w}\;C_{F,x}\left(x\right)-\frac{d\left(D_{F,x}\;C_{F,x}\left(x\right)\right)}{dx}$$

Equation (15) can be integrated with the initial condition ($C_{F,x}\left(0\right)=C_{m,F,x}$) and the boundary condition ($C_{F,x}\left(l_{m}\right)=C_{w,F,x}$). Assuming a constant diffusion coefficient and combining this equation with Equation (14), the solute concentration at the feed membrane wall can be expressed as:

(16) $$C_{w,F,x}=C_{F,x}\;exp\left(J_{w}\;\left(\frac{1}{k_{F}}+\frac{S}{D_{x}}\right)\right)+\frac{J_{x}}{J_{w}}\;\left[1-exp\left(J_{w}\;\left(\frac{1}{k_{F}}+\frac{S}{D_{x}}\right)\right)\right]$$

By contrast, as the direct solute concentration in the DS will be very low, it can be assumed that the ECP can be neglected [27,28] and $C_{w,D,x}=C_{D,x}$.

The mole balance equations for the two solutions can be written as follows:

(17) $$C_{F,x}\;\left(V_{F,o}-J_{w}\;A_{M}\;t\right)=C_{F,x,o}\;V_{F,o}-J_{x}\;A_{M}\;t$$

(18) $$C_{D,x}\;\left(V_{D,o}+J_{w}\;A_{M}\;t\right)=C_{D,x,o}\;V_{D,o}+J_{x}\;A_{M}\;t$$

Equations (12) and (16) can be combined with the mole balance equations (Equations (17) and (18)) to find an individual equation for the direct solute concentration in the bulk and at the membrane walls of both solutions, which can be calculated from the water and direct solute permeabilities, water flux, structural parameter, and mass transfer coefficient at the FS side.

### 2.4. Model Calculations

The concentration at the membrane wall of all solutes present in the FS and DS depends on the water flux, which also depends on these concentrations through the osmotic pressure at the membrane wall. First, the water permeability coefficient, the permeability coefficients of all the solutes, the structural parameter, and the mass transfer coefficients can be fixed to perform the calculation. The water flux at any time can be estimated from the difference between the water flux and the water flux calculated with Equation (1) as follows:

(19) $$J_{w}-L_{p}\;\left(\pi_{w,D}-\pi_{w,F}\right)=0$$

Equation (19) was solved using the software Wolfram Mathematica^®^ 14.0 with the “FindRoot” function and the result obtained was the evolution of the water flux as a function of time. Once this evolution is known, all solute concentrations (inside the membrane and in the bulk solutions) can be calculated over the process time. 

## 3. Materials and Methods

### 3.1. Reagents, Solutions, and Analytical Techniques

All the chemicals used were supplied by Panreac Química S.L.U. (Barcelona, Spain) and were obtained in a pure grade. The pure water used in this study was prepared using a Milli-Q water purification system with a water conductivity less than 10 µS/cm.

The first set of experiments was conducted with two inorganic salts, including NaCl and Na_2_SO_4_ at different concentrations. The DS were prepared by solubilizing the powder salt in water at a global concentration of 2, 1.5, 1, and 0.5 M for NaCl or 1 and 0.5 M for Na_2_SO_4_, to avoid salt precipitation. The study of ammonia permeation was conducted with DS formed with 1.5 M NH_4_Cl or 1.2 M (NH_4_)_2_SO_4_ at an initial pH of 3.5, 6.5, 7.5, 9.5, 10.5, or 11.5, which was fixed by adding small quantities of HCl or NaOH concentrated solution. 

The performance of FO was determined by measuring the solute concentration, conductivity, and pH of the DS and FS, as well as the volume variation in the two chambers. The salt concentration was calculated by measuring conductivity using the HQ40d meter (Hach, Loveland, CO, USA) integrated with an IntelliCAL™ CDC401 Laboratory 4-Poles Graphite Conductivity Cell. The pH of the solution was determined from the IntelliCAL™ CDC and PHC probes connected to an HQ40d multimeter (Hach, Loveland, CO, USA). The TAN content (ammonia and ammonium ion concentrations) was determined using a high-performance ammonia ion selective electrode Orion 9512HPBNWP (Thermo Fisher Scientific Inc., Waltham, MA, USA) following the 4500-NH3D procedure.

### 3.2. Experimental Equipment

**Figure 2:**
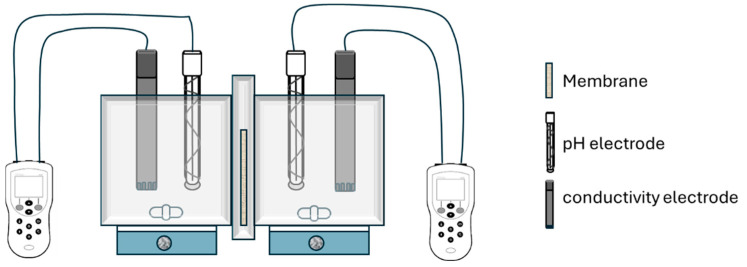
Schematic representation of the dead-end bench-scale cell with the pH and conductivity electrodes.

The experiments were conducted in a dead-end bench-scale cell, which consists of two cubic chambers with a capacity of 1 L that are connected through the 50 cm^2^ FO membrane (Figure 2). The chambers were perfectly sealed to avoid ammonia volatilization and they were agitated vigorously with a magnetic stirrer to minimize the effect of the ECP. One chamber was filled with 0.750 L of the FS and the other chamber was filled with 0.500 L of the DS and the conductivity and pH electrodes were placed in both chambers. All experiments were conducted in the PRO mode, where the membrane active layer was in contact with the DS. 

This study was conducted with the commercial “OsMemTM TFC-ES FO” membrane, supplied by Hydration Technology Inc. (Albany, OR, USA), which is composed of a thin-film composite (TFC) polyamide membrane and an embedded polyester screen support (lot no. 130524). This membrane can operate at a pH range of 2–11 and has a 99.4% salt rejection, as indicated in the test condition manual. Typically, many studies have compared the performance of the TFC and CTA membranes. However, we did not use the CTA membranes because they are not suitable for TAN recovery as the maximum operating pH is 8. All the membrane pieces were soaked in deionized water 2 days prior to their use in the experiment at room temperature. Three membrane pieces, which were used in each experiment and had similar properties since they were manufactured in the same batch of production, were placed in three cells to check the reproducibility of the experimental results. All experiments were performed in triplicate and the results proved to be highly reproducible, with the standard deviations being lower than 10% of the average measurements. The temperature of all the experiments was maintained at 20 ± 2 °C by air conditioning in the summer and heating in the winter. The first experiment (2 M NaCl as the DS and pure water as the FS) was repeated several times in the three cells to verify that the membrane performance was in steady state.

### 3.3. Modeling Procedure

#### 3.3.1. Modeling Variables Calculations

One key point in simulations is the determination of the solute osmotic pressure at the membrane wall, specifically at very high solute concentrations in the DS. In these conditions, the ideal Van’t Hoff equation, which assumes a linear relationship between osmotic pressure and solute concentration, is not valid and the most sophisticated equations have to be used. Therefore, we calculated the osmotic pressure of all the solutes using OLI Stream Analyzer 3.1 (OLI System Inc., Morris Plains, NJ, USA). Table 1 shows the polynomial equations used to calculate the osmotic pressure. In the literature, it was stated that the NaCl concentration at the draw membrane wall does not deviate significantly from the Van’t Hoff equation in the active layer that is in contact with the FS due to the significant effect of the ICP [27]. The solute osmotic pressure at the membrane wall in the FS was calculated with the Van’t Hoff equation due to the low solute concentrations.

Another important consideration for modeling is the determination of the acidic equilibrium constant of the ammonium ion, $K_{NH4}$, which follows the acid–base equilibrium chemical reaction between the ammonium ion and ammonia in a water solution:

(20) $$NH_{4}^{+}\;\overset{\;}{↔}\;NH_{3}+H^{+}$$

For concentrated ammonium solutions, the acidic equilibrium constant of the ammonium ion depends on the activity coefficients and concentrations of the three components as follows:

(21) $$K_{NH4}=\frac{\gamma_{NH3}\;\gamma_{H}}{\gamma_{NH4}}\;\frac{C_{NH3}\;C_{H}}{C_{NH4}}$$

where $\gamma_{NH3}$, $\gamma_{NH4}$ and $\gamma_{H}$ are the activity coefficients and $C_{NH3}$, $C_{NH4}$ and $C_{H}$ are the concentrations of NH_3_, NH_4_^+^ and H+, respectively. As already known, the activity coefficients depend on the ionic strength and pH of the solution, both of which change in the FO process. To simplify the calculations, the acid–base equilibrium constant was calculated using OLI Stream Analyzer 3.1 (OLI System Inc., Morris Plains, NJ, USA) as a function of pH and TAN concentrations. The estimated equation for $pK_{NH4,calc}$ is the following at 20 °C:

(22) $$pK_{NH4,calc}=\left(0.00170\;pH^{3}-0.0612\;pH^{2}+0.734\;pH-2.68\right)\;C_{TAN}+9.40$$

This correlation of the estimated acid–base equilibrium constants, $K_{NH4,calc}$, is valid for the concentrations at the membrane wall and in the bulk draw solutions. Therefore, knowing the pH and TAN concentration, the NH_3_, NH_4_^+^ concentrations can be calculated as:

(23) $$C_{NH4}=\frac{C_{TAN}\;10^{-pH}}{10^{-pH}+K_{NH4,calc}};C_{NH3}=\frac{C_{NH4}\;K_{NH4,calc}}{10^{-pH}}$$

At this point, the global osmotic pressure at the membrane wall can be calculated with Equation (2). For instance, for a DS of NH_4_Cl at pH 9 (fixed using NaOH), the global osmotic pressure can be written as:

(24) $$\pi_{w,D}=\pi_{w,D,NH3}+\pi_{w,D,NH4Cl}+\pi_{w,NaCl}+\pi_{w,D,NaOH}$$

and the hydroxide ion concentration can be calculated from the charge balance equation at the membrane wall as:

(25) $$C_{w,D,NH4}+\frac{K_{w}}{C_{w,D,OH}}+C_{w,D,Na}=C_{w,D,Cl}+C_{w,D,OH}$$

where is $K_{w}$ the water ionization constant. A similar equation can be written for the charge balance in the FS. Moreover, we considered the loss of NH_3_ by volatilization negligible due to the high solubility of NH_3_ in water and the low value of the Henry constant [24]. 

Lastly, the mass transport equations through the ECP and ICP were solved by considering a constant solute diffusivity coefficient. This assumption is usually established to simplify the resolution of differential equations, as shown in the literature. For instance, the diffusion coefficient of NaCl only varies by 3% in a concentration range of 0.05 to 2 M [27]. The diffusivity coefficients used for calculations were: 1.48 × 10^−9^ m^2^/s for NaCl, 0.657 × 10^−9^ m^2^/s for Na_2_SO_4_, 1.77 × 10^−9^ m^2^/s for NH_4_Cl, 0.893 × 10^−9^ m^2^/s for (NH_4_)_2_SO_4_ [29] and 1.86 × 10^−9^ m^2^/s for NH_3_ [30].

#### 3.3.2. Modeling Algorithm

The proposed model depends on several parameters (*L_p_*, *L_s_*, *L_x_*, *S*, *k_F_*, and *k_D_*) depending on the number of solutes. The evolution of the bulk solute concentrations in the DS and FS can occur as a function of time following the procedure described in Section 2. The model parameters can be estimated by matching the experimental data and the calculations derived from the model. The experimental data were the evolution of the feed volume and the bulk concentrations of all the solutes present in the FS and DS over time. First, these experimental data were adjusted by the best polynomial equation to display more detailed data over time, which were the experimental data used in the fitting procedure and identified with the subindex “exp”. The calculated feed volume over time was estimated from the calculated water flux as $V_{F}^{calc}=V_{F,o}-J_{w}\;A_{M}\;t$. Then, after establishing the initial values of the model parameters, the fitting process was followed by minimizing the global root mean square error, $E_{tot}$, between the experimental data and the calculated data. This global error is defined as the sum of the relative errors of feed volume and all bulk solute concentrations in both solutions:

(26) $$E_{tot}=E_{VF}+\sum_{s=1}^{N_{F}}E_{CF,s}+\sum_{s=1}^{N_{D}}E_{CD,s}$$

(27) $$E_{tot}=\frac{\sqrt{\sum_{t}{\left(V_{F}^{exp}-V_{F}^{calc}\right)}^{2}}+\sqrt{\sum_{s=1}^{N_{F}}\sum_{t}{\left(C_{F,s}^{exp}-C_{F,s}^{calc}\right)}^{2}}+\sqrt{\sum_{s=1}^{N_{D}}\sum_{t}{\left(C_{D,s}^{exp}-C_{D,s}^{calc}\right)}^{2}}}{m-1}$$

where m is the number of data points and $N_{F}$ and $N_{D}$ are the number of solutes present in the FS and DS, respectively. The determination of *L_p_*, *L_s_*, *S*, *k_F_*, and *k_D_* can be performed separately with distinct experimental data, minimizing the possible mathematical coupling with the water and solute permeabilities. In this sense, we conducted experiments with different salts and configurations that enabled the determination of these parameters (as will be commented in Section 4). The algorithm was implemented in Wolfram Mathematica^®^ and it was iteratively solved to find the best model parameters that gave the minimum global error. 

## 4. Results and Discussion

### 4.1. Determination of the External Concentration Polarization

**Figure 3:**
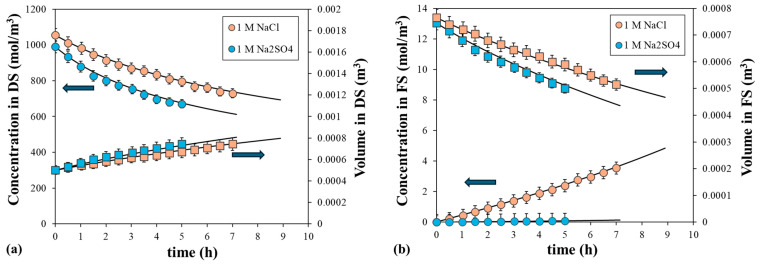
Comparison of the calculated and experimented solute concentrations and volume over time in the (**a**) DS and (**b**) FS, with 1 M NaCl or 1 M Na_2_SO_4_ as the DS and water as the FS. Symbols correspond to the experimental data (squares to volume and circles to solute concentration) and lines to the calculated data. Error bars represent the standard deviation.

The ECP was evaluated by performing experiments in the PRO mode (where the active layer is in contact with the DS), minimizing the impact of the internal polarization. These experiments were carried out with water as the FS and solutions of different types and concentrations of two inorganic salts as the DS. Sodium chloride (NaCl) was used as a model draw solute and sodium sulfate (Na_2_SO_4_) was selected for its expected low solute permeation based on its high molecular weight and ionic charge. Specifically, we analyzed the effect of driving forces on the water flux by using 0.5, 1, 1.5 and 2 M NaCl or 0.5 and 1 M Na_2_SO_4_ as the DS and pure water as the FS in contact with the support layer. Higher Na_2_SO_4_ concentrations were not used to avoid salt precipitation. As an example, Figure 3 shows the evolution of the solute concentrations and volumes over time in the DS and FS for 1 M NaCl or 1 M Na_2_SO_4_ as the DS. Under these conditions, the two solutions had a similar initial osmotic pressure: 46.4 and 45.7 atm, respectively. As expected, the dilutive process of the DS and the concentrative process of the FS were observed due to water permeation caused by the difference in the driving force. The reduction of volume of the FS was due to a decrease in the osmotic pressure difference between the two solutions and possibly due to the impact of the ECP, especially in the DS. It is important to note that the permeation process was faster for Na_2_SO_4_ than for NaCl, instead of the initial osmotic pressure. Thus, the volume of the FS decreased from 0.750 L to 0.500 L after 5 h and after 7 h, respectively. Conversely, the reduction of the driving force, calculated from the initial to the final value at the above times, was similar for both DS, being 18.3% and 19.5% for Na_2_SO_4_ and NaCl, respectively. Therefore, the water flux was affected by the osmotic pressure difference and the type of solute. Additionally, Figure 3 shows the slight increase in solute concentration in the FS due to solute permeation. The NaCl concentration in the FS increased from 0 to 3.55 mol/m^3^ after 7 h with a minimum rejection of 99.51%, while the Na_2_SO_4_ concentration in the FS increased from 0 to 0.083 mol/m^3^ after 5 h with a minimum rejection of 99.99%. Therefore, both solutes showed very low permeability, especially Na_2_SO_4_, which can be considered as showing no permeation.

Figure 3 also shows the fitted curves derived using the model, which are presented as continuous lines. The experimental and the calculated data of solute concentrations and volumes of the DS and FS correlated well. The calculations were conducted with the following assumptions:

- The ICP and ECP in the FS were considered insignificant, as the FS was pure water and there was a low solute permeation observed. Different values of the *S* parameter were applied to the same fitting curve.
- The water permeability coefficient was assumed to be independent of the solute solution; therefore, the same value was set for the two solutions.
- The solute permeability coefficient and the mass transfer coefficient in the DS were also independent of the solute solution.
- The mass transfer coefficient in the DS and FS was set to the same value because the hydrodynamic properties of the two chambers were similar.

The values of the water permeability coefficient, solute permeability coefficient, and mass transfer coefficient that best fit the experimental data are shown in Table 2. The mean water permeability coefficient was 1.01 × 10^−7^ ± 0.0342 × 10^−7^ m^3^/(m^2^ s atm) (with a correlation level of 95%), while the mean solute permeability coefficient was 3.21 × 10^−8^ ± 0.195 × 10^−8^ m/s for NaCl and 9.43 × 10^−10^ ± 0.191 × 10^−10^ m/s for Na_2_SO_4_ (with a correlation level of 95%). Note the different levels of magnitude for the two solutes, which was expected from the experimental data. These estimated values were consistent with those reported in the literature. However, there was considerable variability in the parameter values due to differences in the fitting procedure. Numerous studies have investigated the water permeability coefficient using reverse osmosis (RO) cells, applying varying transmembrane pressures to assess water transport through the membranes. Additionally, researchers have analyzed the FO performance at different temperatures, which has a significant influence on the estimated parameter values. Thus, the water permeability coefficient was reported to range from 1 × 10^−7^ to 7 × 10^−7^ m^3^/(m^2^ s atm) and the NaCl permeability coefficient from 0.9 × 10^−7^ to 4 × 10^−7^ m/s [31,32,33,34,35].

The mean mass transfer coefficient was also different for the two solutes, being 5.23 ± 0.667 µm/s for NaCl and 10.2 ± 1.91 µm/s for Na_2_SO_4_ (with a correlation level of 95%). Therefore, the effect of the ECP in the DS was very significant and caused the difference in water fluxes between the NaCl and Na_2_SO_4_ DS. Some studies have suggested that the water permeability coefficient could depend on the salt concentration [36]. However, they have not considered the presence of the ECP, while the observed water flux reduction at high salt concentrations was attributed only to the decrease in the water permeability coefficient. The difference in the strength of the ECP between the two salts may be due to the difference in the electrostatic interaction of the ions with the membrane surface [37], which could be different for monovalent and divalent ions [38]. The comparison of the fitted and experimental data is shown in the Supplementary Materials, following the same assumptions mentioned above. The model parameters are also shown in Table 2. 

**Figure 4:**
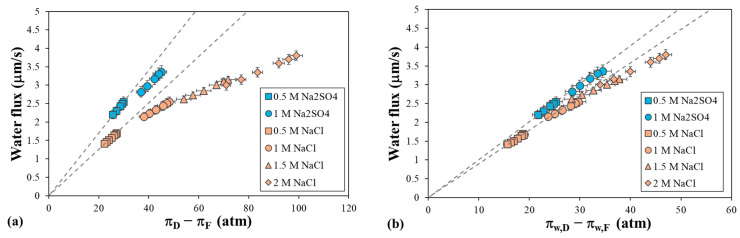
Evolution of the water flux with osmotic pressure difference (**a**) in bulk solutions and (**b**) at membrane walls (driving force) for different DS solutions, with deionized water as the FS. Error bars represent the standard deviation.

Figure 4 shows the water flux at different times (0.2, 0.5, 1, 2, 3, and 4 h) for all the DS tested as a function of osmotic pressure differences. The linear dependence observed for the low salt concentrations is shown as a dashed line. When using Na_2_SO_4_ as the DS, the water flux was observed to be higher than that for NaCl as the DS at a similar range of osmotic pressure differences (Figure 4a). For Na_2_SO_4_, the water flux at 2 h increased from 2.43 to 3.17 μm/s as the DS showed a change in the bulk osmotic pressure difference from 28.6 to 42.3 atm, while for NaCl, the water flux at 2 h increased from 1.57 to 2.44 μm/s as the DS showed an increase in the bulk osmotic pressure difference from 25.0 to 45.7 atm. Similarly, the water flux increased at higher NaCl concentrations, being 3.0 μm/s at 67.1 atm and 3.6 μm/s at 92.0 atm after 2 h. However, as the bulk osmotic pressure difference increased, the water flux behavior was not linear, as it should have been much higher at 2 M NaCl. This could be due to the modification of the water permeability coefficient by the salt concentration or to the significant influence of the ECP. To distinguish between these two effects, we analyzed the water flux as a function of the driving force.

Figure 4b shows that the water flux increased almost linearly with an increasing osmotic pressure difference at the membrane surface, which was calculated by considering the significant effect of the dilutive ECP in the DS. This means that the solute concentration at the membrane surface was much lower than in the bulk solution. The effect of the ECP was more evident at high salt concentrations, such as 2 M NaCl, resulting in an increased water flux that enhanced dilution at the membrane surface [36]. At high salt concentrations, the water flux did not exactly follow a linear relationship. This deviation may indicate a modification in the water permeability coefficient due to the increase in the salt concentration inside the membrane. Other studies have observed this and attributed it to an increase in the significance of the ICP or some osmotic deswelling of the membrane [39]. 

### 4.2. Determination of the Internal Concentration Polarization

The ICP occurs within the porous support layer of the membrane. It leads to an increase in solute concentration near the active layer when operating in PRO mode. Indeed, the ICP is not a selective layer, but it regulates solute permeation by modifying solute diffusivity within the porous structure. Notably, the molecular size of solutes is much smaller than the pore radius of the polyester support layer. Consequently, the water flux is reduced due to the decrease in osmotic pressure difference. The ICP is quantified by the *S* parameter, with smaller values of *S* corresponding to a lower importance of the ICP.

**Figure 5:**
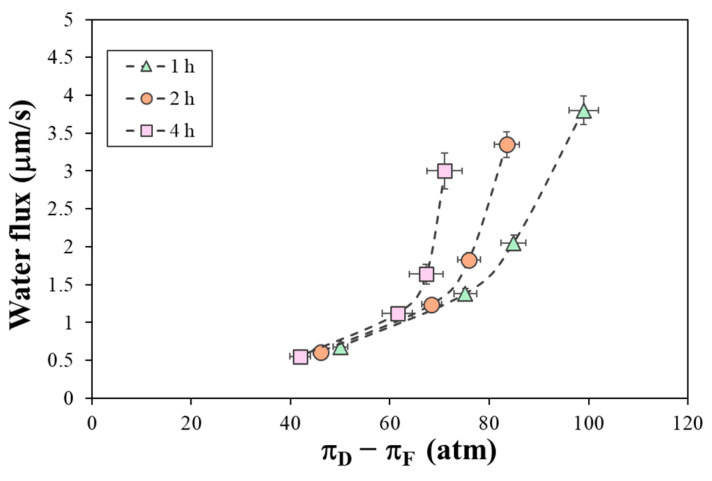
Evolution of water flux as a function of bulk osmotic pressure differences at three different operation times, with 0, 0.25, 0.5, or 1 M NaCl as the FS and 2 M NaCl as the DS. Error bars represent the standard deviation.

The ICP can be assessed by increasing the salt concentration in the FS and positioning the active layer to be in contact with the DS. Consequently, the diffusion of the salt into the porous layer gains significance, thereby altering the osmotic driving force. For this purpose, we conducted experiments exposing the support layer to an increasing NaCl feed concentration (0, 0.25, 0.5, and 1 M), while a 2 M NaCl solution was placed against the active layer. Figure 5 shows the water flux as a function of the bulk osmotic pressure differences at three different operation times. The highest water flux was associated with pure water as the FS, while the bulk osmotic pressure difference decreased significantly over time. As the initial salt concentration in the FS increased, the water flux decreased rapidly non-linearly due to the ICP. In addition, the variation in the osmotic pressure difference with time was less significant. Therefore, the dilutive ICP was primarily responsible for a much lower than expected water flux [36]. Similar results were obtained by Gray et al. [40]. They also analyzed the ICP by placing the active layer in contact with the FS, but they found a linear relationship between bulk osmotic pressure differences and water flux. 

**Figure 6:**
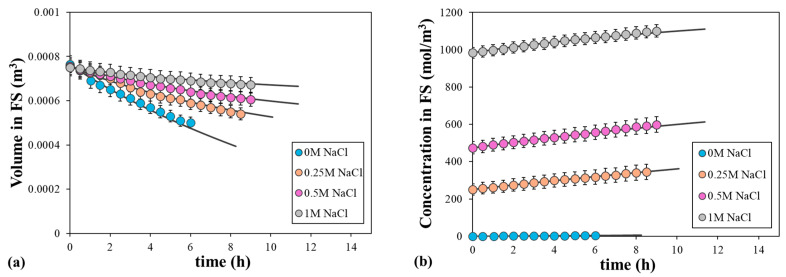
Comparison of the fitted and experimental (**a**) volume and (**b**) solute concentration in the FS, with 0, 0.25, 0.5, or 1 M NaCl as the FS and 2 M NaCl as the DS. Symbols correspond to the experimental data and lines to the calculated data. Error bars represent the standard deviation.

The comparison of the fitted and experimental data is shown in Figure 6, which shows the evolution of salt concentration and volume of the FS over time. The permeation process became slower as the initial salt concentration increased due to the reduction in the driving force. The volume of the FS decreased from 0.750 L to 0.500, 0.590, 0.640, and 0.690 L after 6 h when the NaCl concentrations were 0, 0.25, 0.5, and 1 M, respectively. The fitted data, symbolized by a continuous line, were obtained by simultaneously fitting the data from the four solutions to find the *S* parameter that best adjusted the experimental data, while the permeability coefficients and the mass transfer coefficient were fixed at previously calculated values. In general, the calculated and the experimental data correlated with high agreement (Figure 6), with an *S* parameter value of 565 ± 7.90 μm (with a correlation level of 95%). Only a small deviation between them was observed, especially at a longer time, which may have been due to some variations in the model parameters as the salt concentration inside the membrane increased. This *S* parameter is intrinsic to the physical properties of the membrane support layer, and its value applies to all solutes. The significance of ICP in solute permeation arises from solute diffusivity within the porous support, which relies on both the solute diffusivity and the *S* parameter. The estimated *S* parameter was in line with those reported in the literature, although it depended on the fitting procedure, as was stated above. The reported *S* parameter for the same TFC membrane ranges from 453 to 610 μm [31,35,41,42]. 

### 4.3. Effect of the Initial pH on Water and Solute Permeability Coefficients

**Figure 7:**
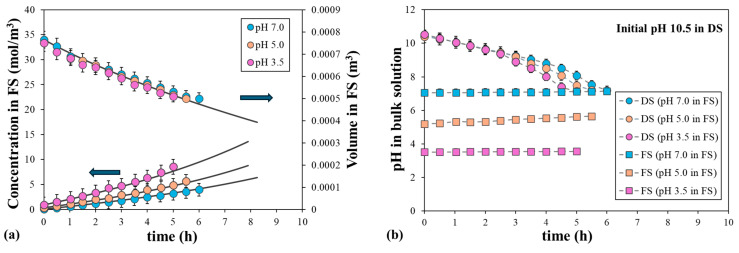
Evolution of (**a**) volume and solute concentration in the FS and (**b**) pH of the bulk FS over time, with 2 M NaCl at pH 10.5 as the DS and water at different initial pH values as the FS. Error bars represent the standard deviation.

Another important variable that can affect membrane performance is the pH of the solution. Therefore, we examined the effect of the pH difference between the FS and DS on water and solute permeability. A 2 M NaCl with an initial pH of 10.5 was used as the DS, while deionized water with an initial pH of 7.0, 5.0, or 3.5 was used as the FS. Figure 7 shows the fitted and experimental values of the volume and NaCl concentration in the FS at these pH values. The volume of the FS decreased with time and this decrease was very similar for the three solutions, indicating that the water flux was almost independent of the pH in the FS. The NaCl concentration increased with time and showed higher values in the FS for the lower initial pH values. The calculated NaCl permeability coefficients were 3.2 × 10^−8^, 3.8 × 10^−8^, and 5.2 × 10^−8^ m/s for the initial pH of 7.0, 5.0, and 3.5, respectively. These different permeability coefficients may be related to the movement of the protons and hydroxyls inside the membrane. 

As it is shown in Figure 7, the pH of the bulk FS remained nearly constant over time, while the pH of the bulk DS decreased significantly from an initial pH of 10.5 to a pH of approximately 7.20 for the three solutions. However, the main difference among them was that the pH of 7.20 was reached after 6, 5.2, and 4.5 h for the initial pH of 7, 5, and 3.5, respectively. This faster decrease in pH of the bulk DS led to enhanced NaCl permeation, likely due to modifications in electrostatic interactions between ions and the membrane surface [43]. Based on the observed improvement in membrane performance, it can be estimated that the membrane’s isoelectric point is approximately pH 3.5, which is consistent with existing literature. Mazlan et al. (2016) [44] measured the zeta potential of the surface of a TFC membrane with 0.5 mM NaCl solution. They estimated the isoelectric point at pH 3.8, which means that the zeta potential was about 5 mV at pH 3.5 and decreased significantly to reach values of −20 mV and −35 mV at pH 5 and 7, respectively. Therefore, the electrostatic interaction between the membrane surface and NaCl decreased with an increasing initial pH in the FS, resulting in higher solute permeation [45].

### 4.4. Determination of Permeability Coefficient of NH_4_^+^ Ion

The permeability coefficient of the NH_4_^+^ ion was analyzed at an acidic pH using two different ammonium salts: NH_4_Cl and (NH_4_)_2_SO_4_. Different initial salt concentrations in the DS were fixed due to the effect of the two salts on the osmotic pressure. That is, 1.5 M NH_4_Cl and 1.2 M (NH_4_)_2_SO_4_ were used at osmotic pressures of 68 and 60 atm, following the OLI calculations. The initial pH of the DS was set to be two units or lower than the pKa of the ammonium–ammonia equilibrium to avoid the presence of ammonia. The pH of the two salts was 3.5, 6.5, and 7.5, which means that the ammonium percentage was 99.99%, 99.93%, and 99.33%, respectively. In all the experiments, deionized water with a pH of 3.5 was used as the initial FS, with the pH fixed by adding HCl or H_2_SO_4_ depending on the salt present in the DS. 

**Figure 8:**
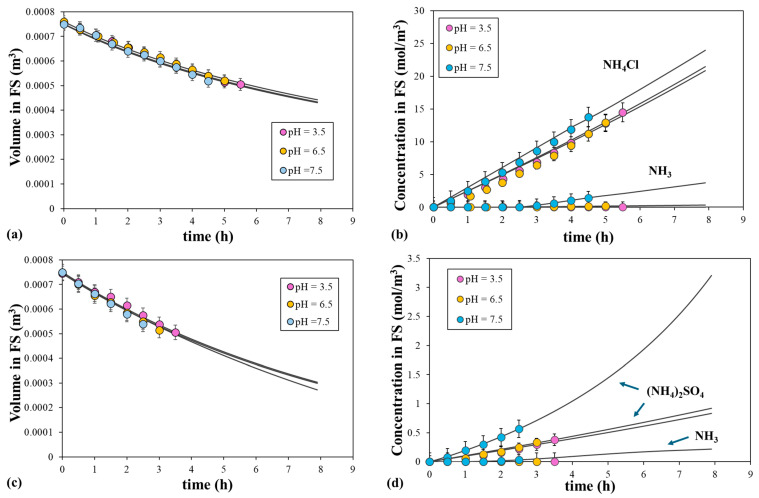
Comparison of the fitted and experimental (**a**) volume and (**b**) solute concentrations in the FS, with 1.5 M NH_4_Cl as the DS, and (**c**) volume and (**d**) solute concentrations in the FS, with 1.2 M (NH_4_)_2_SO_4_ as the DS at pH 3.5, 6.5, or 7.5. Symbols correspond to the experimental data and lines to the calculated data. Error bars represent the standard deviation.

Figure 8 shows the evolution of NH_4_^+^ and NH_3_ concentrations and the volume of the FS as a function of the initial pH of the DS and time for the solutions of the two ammonium salts. Note that the solute concentration for NH_4_Cl was much higher than that for (NH_4_)_2_SO_4_, being 6.85 mol/m^3^ and 0.568 mol/m^3^ at pH 7.5 and 900 s, respectively. These values clearly indicate the different permeation levels of the two salts. At the initial pH of 3.5 and 6.5, the NH_4_Cl concentrations were very similar and the NH_3_ concentration was extremely low. However, the concentration of NH_4_Cl and NH_3_ increased slightly when the initial pH was 7.5. The higher quantity of NH_3_ in the DS at this pH increased the total amount of nitrogen in the FS, with the acid–base equilibrium generating the proportions of ammonium and ammonia. This is also explained by the increase in pH of the FS during the permeation process from 3.5 to 8.6 in 4.5 h, given that the presence of NH_3_ in the FS decreases the proton concentration. A similar pattern was observed for the (NH_4_)_2_SO_4_ solutions, although all the concentrations were very low. On the other hand, the volume of the FS was almost independent of the initial pH of the DS for the two salts. As the water molecules move through the membrane from the FS to DS due to the difference in osmotic pressure, the volume of the FS decreases with time. The reduction in the volume of the FS with time was more abrupt for the (NH_4_)_2_SO_4_ solutions, although the initial osmotic pressure of the two solutions was very similar. For instance, the volume of the FS decreased from 0.750 to 0.500 L in 5.5 h and in 3.5 h for the NH_4_Cl and (NH_4_)_2_SO_4_ solutions at the initial pH of 3.5, respectively. Therefore, the water flux was higher in the presence of sulfate ions, as seen with the sodium salts described above. Almoalimi et al. (2022) [23] found that the ammonium concentration depends on the ion exchange in the DS and ammonium. 

Figure 8 also shows the fitted curves derived from the model. Only the permeation of the ammonium salt at the different initial pH values of the DS was considered. Calculations were conducted using the water permeability coefficient, structural parameter, and mass transfer coefficient values that were determined with the sodium salt. The NH_3_ concentration in the FS was calculated from the pH of the FS and the acid–base constant, which depends on the total TAN concentration and the pH (Equation (22)). As shown in Figure 8, the calculated data were consistent with the experimental data and showed strong correlation for the solute concentrations and the volume of the FS. The mean permeability coefficient values of NH_4_Cl and (NH_4_)_2_SO_4_ were 0.451 ± 0.002 μm/s and 0.0136 ± 0.0005 μm/s, respectively (with a correlation level of 95%). Moreover, both values showed a low standard deviation. As expected, the smaller molecule showed the higher permeability coefficient. Moreover, the ammonium salt concentration in the DS was also fitted well by the model and decreased with time following the dilution of the DS with the water that had permeated from the FS. Therefore, the presence of large-sized and highly negatively charged anions, such as sulphate ions, significantly depleted the transport of ammonium ions. At pH levels above membrane isoelectric point, the negatively charged active layer of the membrane plays a crucial role by enhancing electrostatic repulsion between carboxyl groups in the thin-film composite polyamide membrane and sulphate ions, following the Donnan exclusion principle. Consequently, the rejection of ammonium ions is improved.

### 4.5. Determination of Permeability Coefficient of NH_3_

The determination of the NH_3_ permeability coefficient was complicated since it depends on several parameters. First, it depends on the initial pH of the DS, which determines the concentration of the ammonium ion and ammonia (following the acid–base equilibrium) as well as the concentration of NaCl or Na_2_SO_4_ depending on the ammonium salt used in the DS. Therefore, the water-driven force, as the total osmotic pressure difference at the membrane surface, depends on the osmotic pressure difference of all the solutes present in the two solutions. Moreover, NH_3_ permeation is affected by the ECP and ICP as the solute moves counter-current to the water permeation. Therefore, it is very difficult to obtain a unique permeability coefficient using the typical procedure of FO in the PRO mode. 

**Figure 9:**
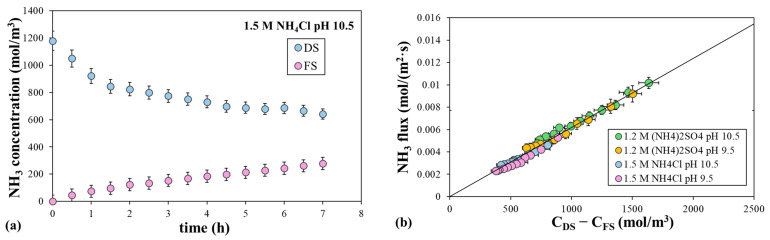
Data from experiments with a null driving force. (**a**) Evolution of NH_3_ concentration in the DS and FS over time, with 1.5 M NH_4_Cl at pH 10.5 as the DS and 1.7 M NaCl as the FS. (**b**) Evolution of the NH_3_ flux with solute concentration difference, with 1.5 M NH_4_Cl or 1.2 M (NH_4_)_2_SO_4_ at different initial pH values as the DS. Error bars represent the standard deviation.

To determine the NH_3_ permeability coefficient without interference from the ICP and ECP, we carried out the experiments by equilibrating the osmotic pressure in the DS with the proper concentration of the sodium salt in the FS, so that the water flux was zero. For instance, 1.7 M NaCl was utilized as the FS to generate a very low mean water flux of 0.0134 µm/s, with 1.5 M NH_4_Cl at pH 10.5 as the DS. The evolution of the NH_3_ concentration in the DS and FS over time is shown in Figure 9. The NH_3_ concentration in the DS decreased from 1180 to 642 mol/m^3^ after 7 h, while it increased in the FS from 0 to 278 mol/m^3^ after 7 h. Similarly, solute rejection decreased almost linearly from 100% to 54.2% after 7 h. Figure 9 also shows the NH_3_ flux as a function of the difference in solute concentration between the DS and FS for the four DS tested and the corresponding sodium salt FS to compensate for the osmotic pressure. The solute flux was calculated from the NH_3_ mols permeated at each time. The NH_3_ flux decreased linearly as the solute driving force decreased for the four solutions. This confirms that the ECP and ICP did not affect the ammonia flux under these conditions. The slope of the adjusted linear equation, using Equation (3), for the solute flux gave a value of 6.18 ± 0.01 µm/s (with a correlation level of 95%) for the NH_3_ permeability coefficient. Note that this value is much higher than the permeability coefficient of sodium chloride and ammonium chloride, demonstrating the strong ability of NH_3_ to permeate the FO membrane. To our knowledge, the NH_3_ permeability coefficient has not been reported in the literature, at least for FO membranes. Zhang et al. (2014) estimated the value of the NH_3_ permeability coefficient from reverse osmosis experiments and found a value within the same order of magnitude as ours [46]. 

The calculated NH_3_ permeability coefficient was checked with the typical FO tests. Thus, experiments were conducted with 1.5 M NH_4_Cl or 1.2 M (NH_4_)_2_SO_4_ at basic pH values as the DS (with higher values of the ammonia/ammonium ratio) and deionized water at an initial pH of 3.5 as the FS. In these conditions, the DS was composed of four solutes that can permeate the membrane and generate osmotic pressure on both sides of the membrane surface. For instance, 1.5 M NH_4_Cl at pH 9.5 generates the following species (calculated with OLI): 0.958 M NH_4_Cl, 0.542 M NH_3_, 0.542 M NaCl, and 2.67 × 10^−5^ M NaOH. As the pH increased, the concentration of NH_4_Cl decreased and the concentration of the other components increased. The NaOH concentration was small compared to the other components, but it modified the ammonium/ammonia ratio, with a small variation in its value. 

**Figure 10:**
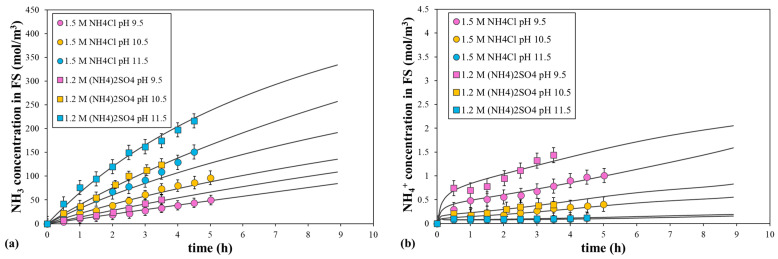
Comparison of the fitted and experimental data for (**a**) NH_3_ and (**b**) NH_4_^+^ concentrations in the FS, with 1.5 M NH_4_Cl or 1.2 M (NH_4_)_2_SO_4_ at different initial pH values as the DS and water as the FS. Symbols correspond to the experimental data and lines to the calculated data. Error bars represent the standard deviation.

Figure 10 shows the ammonia and ammonium salt concentrations in the FS as a function of time for the two DSs at pH 9.5, 10.5, or 11.5. As expected, NH_3_ concentrations were much higher than that of the ammonium salts, with (NH_4_)_2_SO_4_ producing higher ammonia and ammonium concentrations than NH_4_Cl. Moreover, the concentrations of both solutes increased with the initial pH. All these behaviors were associated with a higher initial NH_3_ concentration in the DS for (NH_4_)_2_SO_4_ and an increase in pH. Figure 10 also shows the curves fitted by the model for both solute concentrations, which were obtained by coupling the permeation equations and the acid–base equilibrium reaction at the bulk concentrations in the FS and DS. The calculated data were obtained using the model parameters determined from the ammonium salt, as commented above. In general, the calculated and experimental concentrations correlated with high agreement, demonstrating the viability of the model parameters adjusted previously, especially the NH_3_ permeability coefficient.

**Figure 11:**
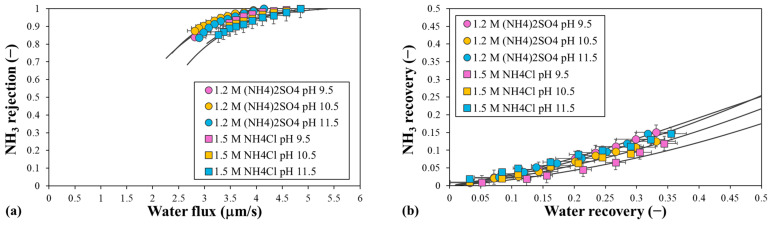
(**a**) NH_3_ rejection with water flux and (**b**) NH_3_ recovery with water recovery, using 1.5 M NH_4_Cl or 1.2 M (NH_4_)_2_SO_4_ at different initial pH values as the DS and water as the FS. Symbols correspond to the experimental data and lines to the calculated data. Error bars represent the standard deviation.

NH_3_ rejection and recovery were also determined for all the experiments. Figure 11 shows the decrease in NH_3_ rejection due to the reduced water flux, as the driving force decreases over time. At the beginning of the test, the rejection was 1 and decreased as the NH_3_ permeated the membrane to reach a mean minimum value of 0.85 at the end of the process. The water flux was always higher for the (NH_4_)_2_SO_4_ solutions, as expected from the results of the sodium salts, mainly due to the effect of the ECP and ICP. Figure 11 also shows the increase in NH_3_ recovery with water recovery and the highest values were obtained at pH 11.5 for the two ammonium salts. The mean NH_3_ recovery was 8%, 10%, and 15% for a water recovery of 35% and a pH of 9.5, 10.5, and 11.5, respectively. A satisfactory correlation was obtained for the NH_3_ rejections and recoveries in all the experiments. The NH_3_ rejections followed a similar evolution with water flux for the solutions with different initial pH values, for each ammonium salt. 

## 5. Conclusions

The determination of the permeability coefficients of the ammonium ion and ammonia depends on the water permeability coefficient, the structural parameter, and the mass transfer coefficients. Therefore, to estimate these parameters, the FO performance in the PRO mode (membrane active layer in contact with the DS) was first analyzed using sodium salts as the DS and deionized water as the FS. The water flux was higher for the Na_2_SO_4_ solutions than for the NaCl solutions. The water permeability coefficient was estimated using the fitting curves for all the DS, resulting in a mean value of 1.01 × 10^−7^ m^3^/(m^2^ s atm). The permeability coefficient of Na_2_SO_4_ was 100 times lower than that of NaCl, mainly due to Donnan’s repulsion at the membrane wall. Conversely, a higher mass transfer coefficient for Na_2_SO_4_ increased the osmotic pressure at the membrane wall, subsequently increasing the water flux. The permeability coefficient of the ammonium ion was estimated at 0.45 μm/s for NH_4_Cl and 0.013 μm/s for (NH_4_)_2_SO_4_ for pH values below 7. Meanwhile, the permeability coefficient of ammonia was determined by fixing the driving force constant at pH levels higher than 9, resulting in an estimated value of 6.18 µm/s. This value was used to fit the experimental data using the model proposed in this study, which considered the ammonium ion–ammonia equilibrium to correct the permeated solute concentrations. The experimental data demonstrated that FO can be used to simultaneously recover both ammonia and water. The maximum NH_3_ recovery was 15% at pH 11.5, with a water recovery of 35%.

## Figures and Tables

**Table 1:** Estimated equations for the calculation of the osmotic pressure for different solutes and concentrations using OLI Stream Analyzer 3.1 (OLI System Inc., Morris Plains, NJ, USA).

Solute	Concentration Range	Osmotic Pressure (atm) *
NaCl	0.2–2 M	$4.14\cdot 10^{-6}\;C^{2}+4.14\cdot 10^{-2}\;C+0.625$
	0–0.2 M	0.0468 C
Na_2_SO_4_	0.2–1 M	$2.43\cdot 10^{-6}\;C^{2}+4.16\cdot 10^{-2}\;C+1.96$
	0–0.2 M	0.0697 C
NH_4_Cl	0.2–1.5 M	$2.70\cdot 10^{-6}\;C^{2}+4.13\cdot 10^{-2}\;C+0.646$
	0–0.2 M	$4.36\cdot 10^{-2}\;C+0.0966$
(NH_4_)_2_SO_4_	0.2–1.2 M	$5.13\cdot 10^{-6}\;C^{2}+4.23\cdot 10^{-2}\;C+1.70$
	0–0.2 M	$4.75\cdot 10^{-2}\;C+0.406$
NaOH	0–0.1 M	0.0454 C

* Concentrations in mol/m^3^.

**Table 2:** Calculated water and solute permeability coefficients and the mass transfer coefficient for the different DS, with deionized water as the FS. Calculations were independent of the selected S parameter.

DS *	Lp × 10^7^ (m^3^/(m^2^ s atm))	Ls × 10^6^ (m/s)	k × 10^6^ (m/s)	Etot (-)(−)
NaCl				
0.5 M	1.01	3.37	4.8	1.53
1 M	0.990	3.24	5.1	2.02
1.5 M	0.985	3.10	5.8	2.55
2 M	0.980	3.13	5.2	1.78
Na_2_SO_4_				
0.5 M	1.06	0.0945	10.0	1.02
1 M	1.04	0.0942	10.3	1.69

* Initial concentration in the DS.

## Data Availability

The data supporting this study’s findings are available upon request from the corresponding author.

## Supplementary Materials

The following supporting information can be downloaded at: https://www.mdpi.com/article/10.3390/polym16131834/s1. Figure S1: Comparison of the calculated and experimented solute concentrations and volume over time in the (a) DS and (b) FS, with 0.5 M NaCl or 0.5 M Na_2_SO_4_ as the DS and water as the FS. Symbols correspond to the experimental data and lines to the calculated data. Figure S2: Comparison of the calculated and experimented solute concentrations and volume over time in the (a) DS and (b) FS, with 1.5 M or 2 M NaCl as the DS and water as the FS. Symbols correspond to the experimental data and lines to the calculated data.

## Nomenclature

Roman symbols	
A_M_	Effective membrane area (m^2^)
C_D,s_	Reverse solute concentration at the DS side (mol/m^3^)
C_F,s_	Reverse solute concentration at the FS side (mol/m^3^)
C_D,s,o_	Initial reverse solute concentration at the DS side (mol/m^3^)
C_F,s,o_	Initial reverse solute concentration at the FS side (mol/m^3^)
C_m,F,s_	Reverse solute concentration at support layer in the FS side (mol/m^3^)
C_w,D,s_	Reverse solute concentration at active layer in the DS side (mol/m^3^)
C_w,D,s_	Reverse solute concentration at active layer in the FS side (mol/m^3^)
C_D,x_	Forward solute concentration at the DS side (mol/m^3^)
C_F,x_	Forward solute concentration at the FS side (mol/m^3^)
C_D,x,o_	Initial forward solute concentration at the DS side (mol/m^3^)
C_F,x,o_	Initial forward solute concentration at the FS side (mol/m^3^)
C_m,F,x_	Forward solute concentration at support layer in the FS side (mol/m^3^)
C_w,D,x_	Forward solute concentration at the active layer in the DS side (mol/m^3^)
C_w,D,x_	Forward solute concentration at the active layer in the FS side (mol/m^3^)
D_D,s_	Reverse solute diffusion coefficient in the DS (m^2^/s)
D_F,s_	Reverse solute diffusion coefficient in the FS (m^2^/s)
D_F,p,s_	Reverse solute diffusion coefficient inside the membrane support layer (m^2^/s)
D_D,x_	Forward solute diffusion coefficient in the DS (m^2^/s)
D_F,x_	Forward solute diffusion coefficient in the FS (m^2^/s)
D_F,p,x_	Forward solute diffusion coefficient inside the membrane support layer (m^2^/s)
J_s_	Reverse solute flux (mol/(m^2^·s))
J_w_	Water flux (m^3^/(m^2^·s))
J_x_	Forward solute flux (mol/(m^2^·s))
k_D_	Solute mass transfer coefficient at the DS side (m/s)
k_F_	Solute mass transfer coefficient at the FS side (m/s)
l_m_	Membrane support layer thickness (m)
L_p_	Water permeability coefficient (m^3^/(m^2^·s·atm))
L_s_	Reverse solute permeability coefficient (m/s)
L_x_	Forward solute permeability coefficient (m/s)
R_m_	Ideal gases constant (8.31 J/(mol·K))
S	Structural parameter (m)
T	Absolute temperature (K)
V_D,o_	Initial Volume of the DS chamber (m^3^)
V_F,o_	Initial Volume of the FS chamber (m^3^)
Greek symbols	
δ	Effective EPC thickness (m)
ε	Membrane support porosity (-) (−)
ϕ_w,D,s_	Osmotic coefficient at the membrane wall in the DS (−)
ϕ_w,F,s_	Osmotic coefficient at the membrane wall in the FS (−)
π_w,D_	Global osmotic pressure at the active layer in the DS (atm)
π_w,F_	Global osmotic pressure at the active layer in the FS (atm)
τ	Membrane support tortuosity (−)
Abbreviations	
DS	Draw Solution
ECP	External Concentration Polarization
FO	Forward Osmosis
FS	Feed Solution
ICP	Internal Concentration Polarization
PRO	Pressure Retarded Osmosis
TAN	Total Ammoniacal Nitrogen
