# Supplementary material for: Viability of Total Ammoniacal Nitrogen Recovery Using a Polymeric Thin-Film Composite Forward Osmosis Membrane: Determination of Ammonia Permeability Coefficient
Source: Polymers (Basel). 2024 Jun 27;16(13):1834. doi: 10.3390/polym16131834 (PMC11244275; doi:10.3390/polym16131834)
Supplement: Supplementary file 1 [file polymers-16-01834-s001.zip › polymers-3053459-supplementary.pdf]

Supplementary Material

Viability of total ammoniacal nitrogen recovery using a polymeric thin-film composite FO membrane: determination of ammonia permeability coefficient

Shirin Shahgodari, Joan Llorens and Jordi Labanda*

Department of Chemical Engineering and Analytical Chemistry, University of Barcelona, Martí i Franquès 1, 08028 Barcelona, Spain

*E-mail: jlabanda@ub.edu

*Fax: 34 934021291

*Telf: 34 934031334

Figure S1

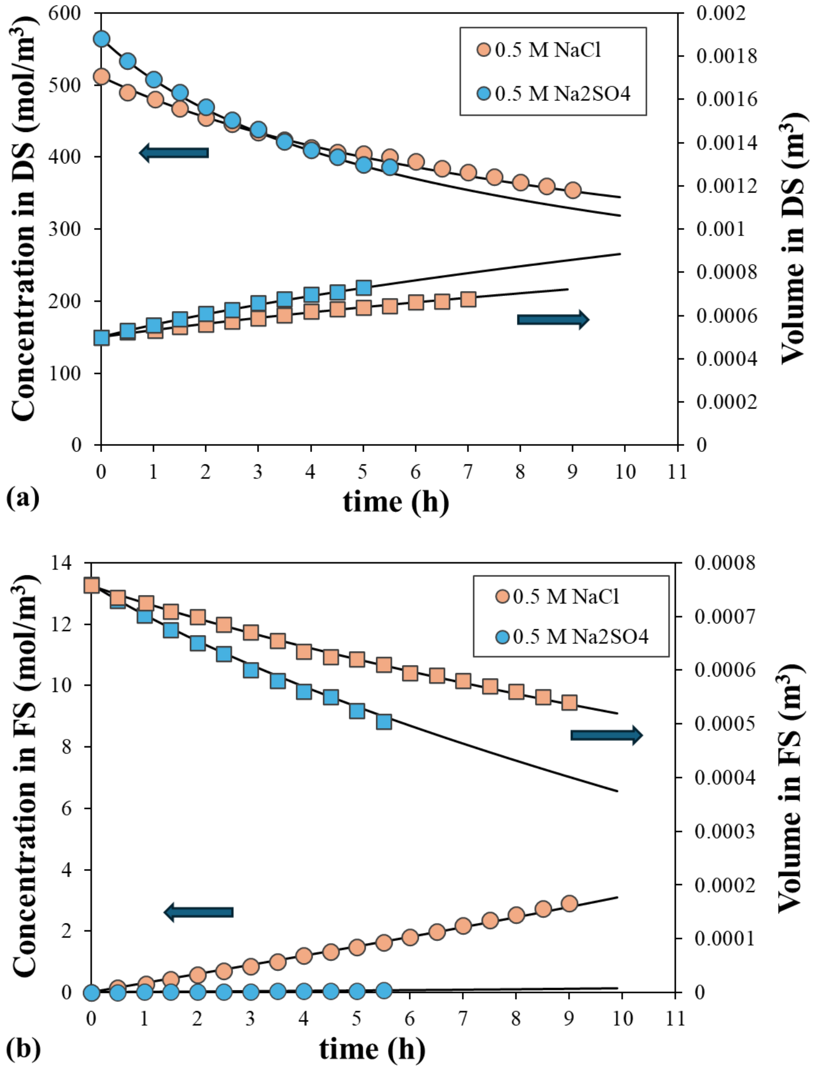

Figure S1. Comparison of the calculated and experimented solute concentrations and volume over time in the (a) DS and (b) FS, with 0.5 M NaCl or 0.5 M Na_2SO_4 as the DS and water as the FS. Symbols correspond to the experimental data and lines to the calculated data.

Figure S2

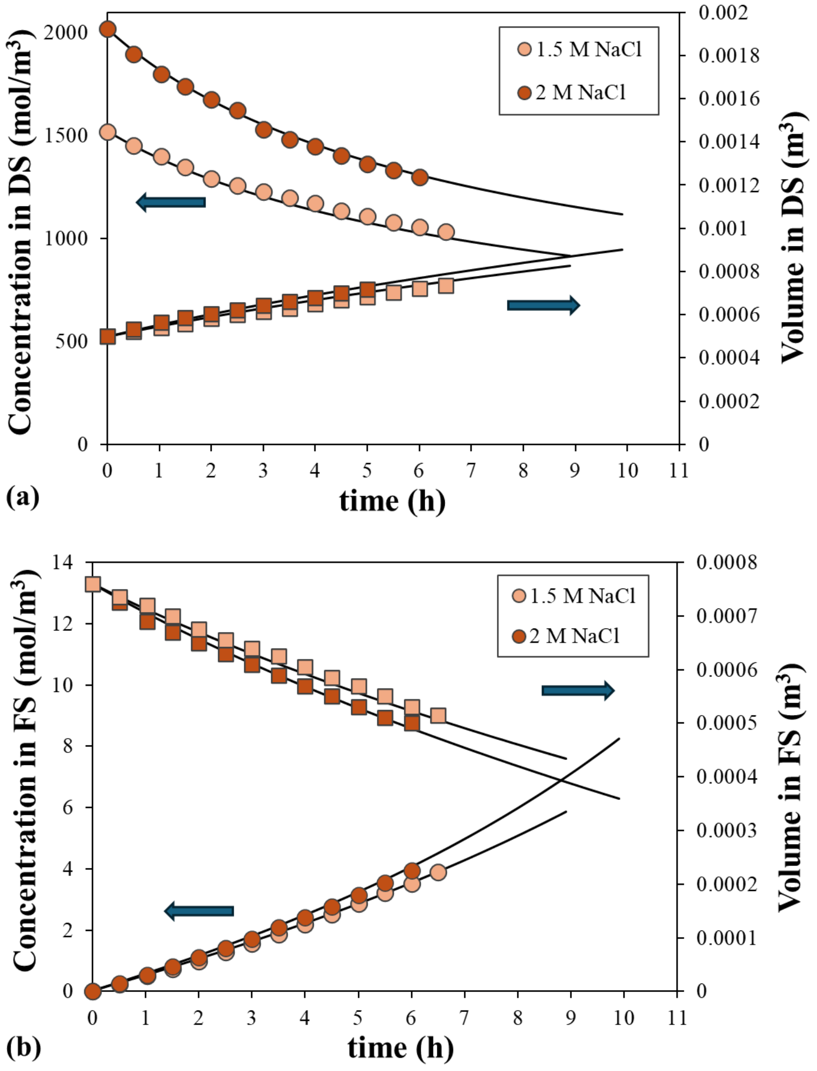

Figure S2. Comparison of the calculated and experimented solute concentrations and volume over time in the (a) DS and (b) FS, with 1.5 M or 2 M NaCl as the DS and water as the FS. Symbols correspond to the experimental data and lines to the calculated data.
